# Role of maraviroc in a dyslipidemic murine model of atherosclerosis RTV-induced

**DOI:** 10.7448/IAS.15.6.18154

**Published:** 2012-11-11

**Authors:** S Cipriani, A Mencarelli, D Francisci, B Renga, E Schiaroli, C D'Amore, S Fiorucci, F Baldelli

**Affiliations:** 1 Università di Perugia, Medicina Sperimentale e Scienze Biochimiche, Perugia, Italy; 2 Università di Perugia, Medicina clinica e sperimentale, Perugia, Italy; 3 Università degli Studi Perugia, Medicina Sperimentale e Scienze Biochimiche, Perugia, Italy; 4 Università degli Studi Perugia, Medicina clinica e sperimentale, Perugia, Italy

## Abstract

**Purpose:**

Chemokines and their receptors play a crucial role in the development of atherosclerosis. CCR5 is considered to be crucial to monocyte recruitment during development of atherosclerosis. CCR5, known as a co-receptor of HIV-1, is the target of the approved CCR5 antagonist maraviroc (MVC). Therefore we investigated whether MVC reduces inflammation and atherosclerosis in a rodent model of dyslipidemia (ApoE-/-mice) treated or not with Ritonavir (RTV).

**Methods:**

Two-month-old mice (8 per group): wild type, ApoE-/- plus vehicle; ApoE-/- plus RTV; ApoE-/- plus RTV in combination with MVC. Nine-month-old mice (13 per group): wild type; Apo E-/- + vehicle; and Apo E-/- + MVC. Animals were sacrificed after 3 months treatments and plasma, aortas and epididymal fat were collected. Areas of aortas plaque were measured. Immunohistochemistry was performed to evaluate macrophages infiltration, and protein levels of cytokines/chemokines (i.e. ICAM, PAI, VCAM, IL-10, IL-17, MCP1, Rantes, TNFα, INFγ) were evaluated in aorta lysates.

**Summary of results:**

RTV enhances the plaque areas percentage in two month old ApoE-/- mice and is significantly reduced by MVC. The ritonavir-enhanced Mac3 expression on plaques is also reduced by MVC. Treatment with MVC lowers aortic concentration of cytokines/chemokines and plasmatic level of CRP that are increased by RTV. Ritonavir, enhancing mRNA expression of IL-6, Rantes and Mip1α, induces lipoatrophy in epididymal fat; MVC revertes this lipoatrophy and reduces mRNA levels of these cytokines-chemokines. Moreover, in ApoE-/- mice 9 months old, MVC significantly reduces the percentage of plaque areas (from 16.6±3.35% to 7.13±1.44%) (en-face coloration), lowers aortic MAC3 staining and reduces the aortic concentration of cytokines/chemokines.

**Conclusions:**

In a dyslipidemic rodent model the CCR5 inhibitor Maraviroc significantly reduces the percentage of aortic plaque areas, aortic inflammation and lipoatrophy of the epididymal fat. Therefore, the current use of CCR5 antagonists to treat HIV infection, a condition associated with an increased occurrence of cardiovascular disease, should also be exploited to determine any beneficial cardiovascular effects [1,2].

## References

[1] Mencarelli A, Cipriani S, Renga B, Francisci D, Palladino G, Distrutti E (2010). The bile acid sensor FXR protects against dyslipidemia and aortic plaques development induced by the HIV protease inhibitor ritonavir in mice. PLoS One.

[2] Veillard NR, Kwak B, Pelli G, Mulhaupt F, James RW, Proudfoot AE (2004). Antagonism of RANTES receptors reduces atherosclerotic plaque formation in mice. Circ Res.

